# Preliminary Evidence on Psychophysiological and Emotional Responses to Different Types of Human–Calf Interaction in Healthy Adults

**DOI:** 10.3390/ani16162448

**Published:** 2026-08-07

**Authors:** Jae-Sung Lee, Seok-Hyeong Kang, Risu Kim, Sin-Ae Park, Hong-Gu Lee

**Affiliations:** 1 Department of Animal Science, Konkuk University, Seoul 05029, Republic of Korea; jslee78@konkuk.ac.kr (J.-S.L.); kshmilk@seojeong.ac.kr (S.-H.K.); 2 Department of Bio and Healing Convergence, Graduate School, Konkuk University, Seoul 05029, Republic of Koreasapark42@konkuk.ac.kr (S.-A.P.); 3 Department of Forestry and Landscape Architecture, Konkuk University, Seoul 05029, Republic of Korea; 4 Digital Humanities Agro-Healing Convergence Research Center, Konkuk University, Seoul 05029, Republic of Korea

**Keywords:** animal-assisted activity, electroencephalogram, emotional response, dairy calf

## Abstract

This study investigated the effects of human–calf interactions on psychophysiological and emotional responses in humans. Thirty healthy adults (mean age: 25 ± 1.3 years) participated in four distinct 3 min activities with a Holstein calf: (1) watching a 2D video of a calf (video observation), (2) observing a calf directly (direct observation), (3) feeding a calf, and (4) grooming a calf. Electroencephalogram (EEG) measurements were recorded from the prefrontal, frontal, parietal, and occipital lobes during each activity. Participants’ emotional states were assessed using the Profile of Mood States (POMS) questionnaire before and after the sessions. Results showed that relative alpha power spectra, indicative of relaxation and resting states, significantly increased during both direct and video observation of the calf compared to feeding or grooming activities (*p* < 0.05). Relative beta power, associated with concentration and alertness without stress, was also elevated during the observation conditions relative to grooming (*p* = 0.044). Furthermore, POMS scores revealed a significant reduction in total mood disturbance after calf-related activities, particularly following observation (both video and direct) and grooming (*p* = 0.004). These findings suggest that brief interactions with calves may promote relaxation, enhance attention, reduce stress, and improve emotional well-being in healthy adults. The results provide preliminary evidence that may inform future research on the development of animal-assisted activity programs.

## Simple Summary

Human–animal interactions are increasingly recognized as a natural way to reduce stress and support mental health. This study examined whether short interactions with a Holstein calf could improve human brain activity and emotional states. Thirty healthy young adults (mean age: 25 years) participated in four 3 min activities: viewing a video of a calf, observing a live calf, feeding a calf, and grooming a calf. Brain waves related to relaxation and concentration were measured, and emotional states were assessed using the Profile of Mood States questionnaire before and after the activities. The results showed that observing a calf, either on video or in person, most strongly increased brain waves associated with relaxation. All activities reduced negative mood, with observation yielding the greatest benefits for emotional well-being. This study demonstrates that structured calf interactions can promote relaxation and improve mood. These findings provide scientific support for using calf-assisted activities in animal-assisted therapy programs as a natural approach to mental health support.

## 1. Introduction

Global mental health challenges and aging are increasing the risk of dementia worldwide. In South Korea, where suicide rates have remained relatively high in recent years, preventive, nonpharmacological approaches that address psychological, physiological, and emotional well-being through complementary therapies are urgently needed [1]. Mental health in South Korea has emerged as a notable public concern, as evidenced by the following factors: rising suicide rates, increasing prevalence of depression, rising treatment costs for mood disorders, growing numbers of elderly individuals diagnosed with dementia, an increase in crimes associated with mental illness, and a higher incidence of attention-deficit hyperactivity disorder. In response, the South Korean government has established a national objective to reduce the suicide rate by 30% by 2027 [1]. According to the report of the Central Dementia Center of Korea (2023) [2], the number of individuals aged 65 years or older diagnosed with dementia is projected to increase to 1,084,000 (10.32%) by 2025 and 3,027,000 (16.09%) by 2050. Although pharmacological treatments exist, they often fail to achieve full recovery and may cause adverse side effects. Consequently, demand has grown for complementary therapies—especially those involving psychological, physiological, and emotional interactions—highlighting the increasing recognition of nonpharmacological healing methods.

Recent advancements in animal-assisted therapy (AAT) have demonstrated the efficacy of animal–human interactions in managing various diagnosed psychological conditions, with minimal or no reliance on medication [3]. The psychological benefits of animal–human relationships have been extensively documented. Philosophers and scientists have long examined the dynamic and interdependent nature of these associations [4,5]. Although canines and felines are the most frequently utilized animals in animal-assisted activities (AAAs), other species, including but not limited to equines, birds, rabbits, hamsters, ducks, and dolphins, are also employed. Among these species, horses are frequently regarded as particularly effective owing to their capacity for nonverbal communication and profound emotional bonding. Previous studies involving dogs, cats, and horses have reported beneficial effects on a variety of outcomes, including anxiety, depressive symptoms, quality of life, social functioning, and physiological stress-related responses [6,7,8]. In particular, psychophysiological studies of human–dog interaction have demonstrated favorable changes in mood, stress, and EEG-related responses, while equine-assisted interventions have also shown positive effects on psychological and physiological well-being [7,8].

Among farm animals used for AAAs, dairy cows (*Bos taurus* and *B. indicus*) have played an integral role in human civilization since their domestication approximately 8000–10,000 years ago. The domestication of cows marked a major turning point in the development of human agriculture by providing dependable resources such as milk, meat, labor, and hides [9]. More recently, interest in farm animal-assisted interventions has increased, although scientific evidence remains limited compared with that for companion animals and horses [10]. Calves may be appropriate candidates for animal-assisted activities because cattle are social animals capable of establishing constructive relationships with humans, and positive human contact has been associated with reduced stress-related reactivity and calmer behavioral expression. In therapeutic environments, calves may also provide distinctive sensory and emotional experiences through their warmth, soft coat, and relatively gentle temperament. Furthermore, compared with fully grown cattle, calves may be more feasible to incorporate into structured farm-based interventions due to their greater manageability while still preserving the essential qualities of bovine interaction.

In a cognitive analysis of cow-hugging and cow-mediated activities, participants reflected on their experiences and provided feedback during post-session interviews. When asked to share their impressions of cows as companion animals, most participants responded positively [10]. However, this study exclusively examined emotional responses and lacked scientific data, including electroencephalogram (EEG) measurements, to substantiate the psychophysiological effects. Additionally, a scientifically validated study has yet to be conducted in South Korea to demonstrate the healing effects of AAAs using farm animals. Therefore, we aimed to evaluate the psychophysiological and emotional effects of human–Holstein calf interaction across AAA types by EEG measurements and emotional questionnaires.

## 2. Materials and Methods

### 2.1. Participants

Thirty adults aged 20–40 years (16 men, 14 women; mean age: 25 ± 1.3 years) were enrolled between 24 May and 1 June 2024. Recruitment was carried out through notices posted on university bulletin boards in South Korea. Only individuals without allergies to animals and without previous direct contact experience with calves were eligible for participation. Exclusion criteria included a history of cardiovascular disease, such as hypertension, unstable angina, myocardial infarction, or prior cardiac surgery; the presence of psychiatric disorders; use of related medications; and pregnancy or lactation. Prior to participation, all individuals received a full explanation of the study procedures and precautions, and written informed consent was obtained from each participant. Additionally, a demographic questionnaire was administered, encompassing inquiries regarding age and sex.

This study was reviewed and approved by the Institutional Bioethics Committee of Konkuk University (approval no. HR-733). All collected data were coded and stored in anonymized form, and the authors had no access to personally identifiable information after data collection.

### 2.2. Farm Animals

A 10-week-old female Holstein calf was used for the study. Before the beginning of the study, the calf was vaccinated, and its health status was assessed by veterinary examination. The calf was raised under routine farm management conditions and had regular exposure to humans through daily husbandry and handling before the study. However, it had not undergone any specialized training for therapeutic or experimental interactions. A trained bovine handler remained present throughout the session to manage the calf and ensure participant safety in the event of an unexpected situation. After consulting with the handler, it was decided that interacting with the calf five times a day would help minimize its stress levels, considering its health condition. Accordingly, sessions were limited to five per day, each lasting approximately 60 min. No invasive procedures or pharmacological interventions were involved in this study. The study protocol was approved by the Institutional Animal Care and Use Committee in South Korea (KU24079), and all sessions were conducted in accordance with the guidelines of the International Association for Human–Animal Interaction Organizations [11].

### 2.3. Protocol of Farm Animal-Assisted Activities

The experiment was performed in an independent space (2.5 × 6.5 m) at KU farm (Chungju City, Republic of Korea). The space was adequate for the activity, with the handler on standby. It was quiet and insulated from external noise to minimize potential distractions.

**Figure 1:**
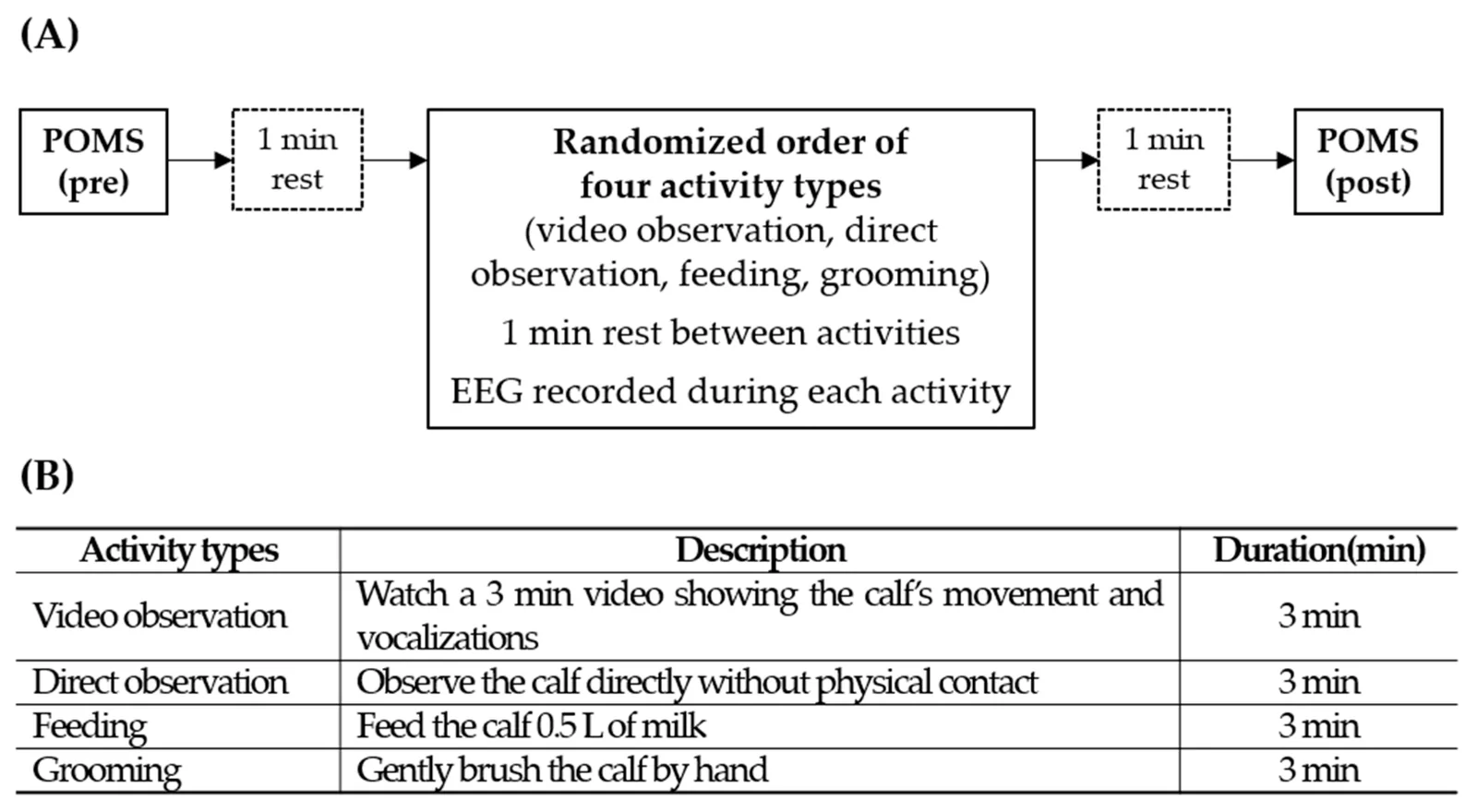
Experimental design of the animal-assisted activity (AAA) protocol: (**A**) Experimental timeline, including POMS assessment, randomized activity order, and resting intervals. (**B**) Description of four activity types: video observation, direct observation, feeding, and grooming.

The participants completed the experimental protocol (Figure 1). Four AAAs were performed for each participant in a single session: (1) video observation, (2) direct observation, (3) feeding, and (4) grooming (Figure 1B). To minimize order effects, the sequence of the four activity conditions was determined using a pre-prepared randomized order list, and each participant completed the activities according to the assigned sequence.

These activities were selected to emphasize direct interaction with the calf and to reflect routine forms of contact commonly observed between humans and farm animals. Before each activity, participants underwent a 1 min resting period, during which they remained seated and looked at a plain wall to minimize external stimulation. The experimental protocol for the activities was designed to consider the calf’s mood in adapting to the stranger and the participants’ mood in accessing the calf. EEG signals were recorded for 3 min during each condition, and participants were instructed to remain silent and avoid sudden movements. Each activity was demonstrated in detail before the experiment. Participants had 4–5 min to complete a questionnaire at the start and end of the experiment, in which they reported their subjective emotional state using the Profile of Mood States (POMS). A 1 min rest period was observed before starting the next activity. After completing the four activities, the experiment was terminated. All the procedures, including four activities, were completed in less than 60 min. During data collection, no intended or unfavorable situations were observed.

### 2.4. Electroencephalogram and Subjective Mood Assessment

EEG activity during the AAAs was recorded using a wireless EEG system (Quick-8; Cognionics, San Diego, CA, USA). The EEG, a non-invasive technique, uses scalp electrodes to accurately and instantaneously measure the brain’s electrical activity [12]. The system uses dry electrodes, allowing rapid removal if a participant experiences discomfort and reducing the safety concerns associated with gel-based wet electrode systems. This device has been widely applied in neuroscience research and meets safety standards established by the European Commission and the Federal Communications Commission. The data were recorded using EEG software (Version 1.0, Bioteck Analysis Software, Daejeon, Republic of Korea). EEG signals were obtained from eight scalp sites: the left and right prefrontal regions (Fp1 and Fp2), left and right frontal regions (F3 and F4), left and right parietal regions (P3 and P4), and left and right occipital regions (O1 and O2). A1 on the left earlobe was used as the reference electrode according to the international 10–20 system [13]. Once the data were collected, following a previous study [14], the EEG data were analyzed using Bioteck software (Version 1.0, Bioteck; Daejeon, Republic of Korea) (Table 1).

The POMS questionnaire was administered at two time points only, namely before the first activity and after the final activity, to evaluate the overall emotional effect of the entire experimental session. The POMS has been used since 2003 by McNair et al. [15]. The Korean version of the POMS, translated by Yeun and Park (2006) [16], was used here. The POMS consists of 65 items grouped into six domains: fatigue–inertia (F), depression–dejection (D), tension–anxiety (T), anger–hostility (A), confusion–bewilderment (C), and vigor (V). This instrument was used to evaluate the participants’ current emotional condition. For each item, participants rated the extent to which the described feeling reflected their present state on a 4-point scale ranging from 1 (“not at all”) to 4 (“extremely”). Based on the responses, a total mood disturbance (TMD) score is calculated by summing the scores of each subcategory (F + D + T + A + C − V). A low score indicates a positive emotional state [15,16].

### 2.5. Statistical Analysis

All statistical analyses were performed using JMP Pro version 17 for Windows (JMP Statistical Discovery LLC, Cary, NC, USA). As all participants were exposed to each of the four activity conditions, the EEG data had a within-subject repeated-measures structure. Therefore, differences among activity conditions were assessed using one-factor repeated-measures ANOVA, with activity type treated as the within-subject factor, followed by Tukey’s honestly significant difference test for post hoc comparisons where appropriate. For subjective mood assessment, POMS scores measured before and after completion of the entire experimental session were compared using Student’s *t*-test. Data are presented as mean ± standard deviation, and *p*-values < 0.05 were considered statistically significant.

## 3. Results

The study included 30 adults aged 20–40 years (mean age 25 ± 1.3 years). Sixteen participants were male (25 ± 2.2 years), and 14 were female (24 ± 1.3 years). No statistically significant age differences were observed between sexes (*p* = 0.448).

Changes in POMS scores were analyzed across six domains: fatigue–inertia, depression–dejection, tension–anxiety, anger–hostility, confusion–bewilderment, and vigor. Significant decreases were observed in tension–anxiety (*p* = 0.006), depression–dejection (*p* = 0.040), fatigue–inertia (*p* = 0.005), and confusion–bewilderment (*p* = 0.017) after the AAAs compared to baseline values. By contrast, anger–hostility and vigor did not show significant changes (*p* = 0.179). The overall TMD score was also significantly reduced after participation in the calf-related activities, including watching a 2D movie (video observation), watching a calf (direct observation), feeding, and grooming, indicating an overall improvement in mood state (*p* = 0.004) (Table 2).

The RA power spectrum analysis revealed that activity in the bilateral parietal (*p* < 0.001) and occipital (*p* < 0.001) lobes and the right frontal (*p* = 0.021) lobe markedly increased during video observation. A markedly increased activity was also observed in the parietal lobes during direct observation (*p* < 0.001). The RFA power spectrum analysis revealed markedly higher results during video observation on both sides of the parietal (*p* < 0.001) and occipital (*p <* 0.001) lobes and the right frontal (*p* = 0.014) lobe compared to the other activities. The ratio of alpha to high-beta (RAHB) analysis revealed that video observation was associated with higher activation in both the left and right parietal lobes compared with feeding or grooming (left: *p* = 0.005; right: *p* < 0.001). Direct observation exerted a more selective effect, as a significant difference was detected only in the right parietal lobe (*p* < 0.001). No comparable differences were observed in the left parietal lobe. Comparing the RA, RFA, and RAHB power spectra revealed a low level of grooming compared to that of video observation. The RB power spectrum analysis revealed that grooming activity was associated with lower activation in the left frontal lobe (*p* = 0.044). In contrast, a marked increase in activity was observed in the left frontal lobe during video observation and direct observation (*p* = 0.04). No differences were observed in the parietal and occipital lobes in the RB power spectrum for any activity. The RLB index revealed that video observation induced a marked increase in brain activity in both the left and right parietal lobes compared with other activity conditions (left: *p* = 0.021; right: *p* = 0.012). Additionally, video observation was associated with heightened activation in the right occipital lobe (*p* = 0.029) (Table 3).

## 4. Discussion

This study examined psychophysiological and emotional responses to different types of AAA. EEG data were collected from healthy adults engaged in various activities with a Holstein calf. The results indicated that distinct brainwave patterns were elicited depending on the activity performed.

Observing a calf’s movement on screen (video observation) resulted in increased brain activity in the RA and RFA power spectrum indices of the frontal lobe. According to previous studies, increased alpha power reflects relaxation and emotional stability [17]. In addition, greater alpha activity has been linked to enhanced cognitive performance, including memory-related processes, as well as lower levels of mental stress [18,19]. RFA is generally interpreted as an index of focused attention maintained under relatively relaxed conditions [20,21]. Higher RFA values may therefore reflect a stable and alert brain state that supports cognitive processing, such as mental rotation tasks. Furthermore, increased RFA is correlated with cognitive judgment, learning ability, and creative thinking [22]. The RAHB is the ratio of alpha waves to high-beta waves and is related to stability and relaxation. High RAHB values indicate high stability and relaxation, whereas lower values mean more stress or arousal [23]. Consequently, these findings may provide a preliminary basis for future research on farm animal-based AAA programs aimed at supporting emotional relaxation and stress management. The frontal lobe includes the motor and prefrontal regions and is known to contribute to the regulation of voluntary movement, behavioral responses, and a variety of higher cognitive functions. In particular, this region is closely associated with attentional control, language processing, problem-solving, and decision-making, which suggests that changes in frontal activity may reflect alterations in cognitive engagement and behavioral regulation during animal-assisted activities [24,25]. The parietal lobe, which makes up approximately 10–15% of the cerebral cortex, is responsible for integrating sensory information, perceiving space, regulating movement direction, and focusing. This study demonstrated that this region processes sensory information, including touch, temperature, and pain, and facilitates the recognition of body position and movement [26]. Collectively, these findings indicate that the activation of the frontal and parietal lobes is associated with cognitive function, suggesting enhanced attention. Therefore, we assume that increased RA, RFA, and RAHB power in the frontal and parietal lobes during video observation or direct observation of a calf implies improved relaxation and emotional stability (Table 3). This result agrees with those of previous studies that found that increased alpha waves in these regions may help reduce stress and promote relaxation when observing animals in a safe environment or that being around nature or animals can produce similar relaxation [27,28,29]. Furthermore, this result is consistent with previous findings from a horseback-riding intervention study, in which RFA power increased after the riding program relative to the control group [21,30]. Several studies have revealed that interactions with canines in AAA settings elevate oxytocin concentrations [31,32,33]. Other studies have also demonstrated a correlation between friendly interactions with canines and reduced cortisol concentrations [31,34]. Hormonal markers such as cortisol and oxytocin provide objective indicators of stress-related physiological activity involving the endocrine and autonomic nervous systems. Because psychological processes can influence bodily function and shape emotional experience [35], these mechanisms help explain the patterns observed in our subjective mood assessments. The POMS results revealed that all participants experienced markedly reduced “tension–anxiety” after the AAA program with the calf (Table 2). This study suggests that human–Holstein calf interactions can lead to emotional stability and decreased mental stress, as indicated by an increase in the alpha power spectrum in the frontal and parietal lobes.

The frontal or parietal lobes’ RB power spectrum index revealed heightened brain activity during video observation or direct observation (Table 3). Previous studies have shown that increased beta-band power reflects an alert and attentive brain state and is associated with focused cognitive processing and motor activity [36,37,38]. The RLB power spectrum index revealed markedly higher brain activity during video observation in three channels of the parietal and occipital lobes. Located between the frontal and occipital lobes, the parietal lobe plays an important role in integrating sensory input from different parts of the body, processing external stimuli, spatial orientation, and motor regulation [25,39]. Located in the posterior part of the cerebral cortex, the occipital region is primarily involved in visual processing [40]. This region is activated by visual input and serves as a major center for receiving and transmitting visual information [41]. Activation in the parietal and occipital lobes during video observation, as an indirect experience, suggests that the participants focus on the calf’s movement and appearance before performing the other activities. RLB activity has been described within the sensorimotor cortex and is commonly referred to as the sensorimotor rhythm [42]. This rhythm has been associated with cognitive performance, including reaction time, psychomotor function, and spatial processing [43,44]. Increased RLB is generally observed during states of relaxed attention in the absence of marked stress [45,46]. Hence, these activities can be selected for farm animal-based AAA programs for patients who expect beneficial health effects derived from increased concentration. Increased RB and RLB indices during video observation or direct observation may facilitate the enhancement or maintenance of attention and concentration in the absence of stress. Our findings are consistent with those of earlier research on the use of AAT following pediatric surgical procedures. Pediatric patients who participated in sessions with a therapy dog exhibited elevated beta activity in their EEG [47]. Another study found that frontal lobe activity was higher when participants interacted with live animals than when they interacted with stuffed animals. In addition, stronger contact with the animal was associated with greater frontal lobe activation [48]. A comparison of our results with those of previous studies reveals a lack of correspondence. Brain activity in the RLB power index during video observation and direct observation increased as the intensity of contact with the animal decreased. This phenomenon can be attributed to the limited and inadequate contact experience with calves that participants had through their engagement with a one-time AAA program. We suggest that sustained interactions with calves would enhance participants’ beta activity. However, POMS evaluations revealed that participants reported feeling remarkably less “confusion–bewilderment” when they engaged in the farm animal-based AAA program. Based on these results, it is necessary to develop a farm animal-based AAA program that maximizes the positive health effects through enhanced concentration.

Differences across the four activity types (video observation, direct observation, feeding, and grooming) in our EEG and POMS outcomes are consistent with three established psychosocial mechanisms. First, according to Serpell’s affect–utility model [49], when an animal is reframed from a production object to a recipient of non-utilitarian contact, affective engagement is reinforced. This explains why the indices of cortical relaxation rose most under video observation and direct observation, where the calf was a passive visual object of attention. Second, the meat-paradox framework [50,51] shows that humans suppress the perceived sentience of animals with high food-association; this attenuation is mediated by perceived sentience and phylogenetic proximity and is consistent with the weaker relaxation signature we observed during feeding and grooming. Third, individual differences in familiarity with animals and demographic background [52,53] modulate these appraisals; we therefore restricted the present sample to healthy young adults without precedent farm-animal contact so that the activity manipulation—rather than participant characteristics—would drive the observed differences.

Several limitations of the present study should be noted. First, the sample included both pet owners and non-pet owners, and prior experience with animals or personal attitudes toward them may have influenced individual responses to the AAA. Participants who were more familiar with animals or more comfortable interacting with them may have shown more favorable responses, which could have affected the results. Second, the relatively small sample size may limit the robustness and generalizability of the findings. Because this study was designed as an initial exploratory investigation, the present results should be interpreted with caution. Future studies with larger and more diverse samples are needed to confirm the robustness and external validity of the observed effects. In addition, research on the neurophysiological mechanisms underlying human–animal interaction remains limited. Therefore, further studies with larger and more diverse populations are needed to confirm the effects of AAAs on brain activity and emotional responses. In addition, the present study did not include circulating biomarkers that could complement the EEG-based indices of relaxation and attention. Future studies should incorporate the measurement of cortisol, oxytocin, and secretory immunoglobulin A, which would provide valuable physiological evidence to further elucidate the mechanisms underlying the psychophysiological and emotional responses observed during video observation, direct observation, feeding, and grooming with calves. Such biomarker-integrated designs would also enhance the statistical robustness and external validity of future AAA investigations involving farm animals. Furthermore, the present study did not include calf-side welfare indicators (e.g., lying/ruminating behavior, postural change, vocalization, eye-white percentage, or autonomic markers) during the four human–calf activities; future studies adopting a One Welfare framework [54]—in which human psychophysiological outcomes and calf welfare state are measured simultaneously—will be required to test the bidirectional hypothesis that calf welfare state and human psychophysiological response are co-constructed during animal-assisted activities.

## 5. Conclusions

The present study demonstrated that AAAs involving a calf were linked to changes in brain activity in healthy adults. In particular, video observation and direct observation appeared to facilitate neural responses associated with relaxation and focused attention, while feeding and grooming elicited different patterns of brain activity that were less consistent with the relaxation profile. Subjective mood assessment also suggested that calf interaction contributed to reduced stress and more positive emotional states. These findings offer practical support for the design of AAA programs and may assist in identifying which of the four activity types—video observation, direct observation, feeding, and grooming—is best suited to different participant groups. Additional studies are warranted to replicate these findings and to further clarify the associations between the four activity types and brainwave patterns. A better understanding of these relationships will help elucidate the mechanisms through which human–farm animal interactions affect psychological and physiological responses.

## Figures and Tables

**Table 1:** EEG power spectrum indices analyzed in this study.

Abbreviation	EEG Index	Frequency Range (Hz)
RA	Relative alpha	8–13
RFA	Relative fast alpha	11–13
RAHB	Ratio of alpha to high beta	8–13/20–30
RB	Relative beta	13–30
RLB	Relative low beta	12–15

**Table 2:** Changes in Profile of Mood States (POMS) scores before and after the animal-assisted activities.

Variable	POMS		
	Pre-Activity	Post-Activity	*p*-Value
Tension–anxiety (T)	7.0 ± 0.95	4.0 ± 0.48	0.006 *
Depression–dejection (D)	3.1 ± 0.69	1.4 ± 0.43	0.040 *
Anger–hostility (A)	1.0 ± 0.51	0.3 ± 0.17	0.179
Vigor (V)	28.3 ± 2.21	32.3 ± 2.66	0.252
Fatigue–inertia (F)	6.0 ± 0.90	2.8 ± 0.61	0.005 *
Confusion–bewilderment (C)	5.6 ± 0.50	3.9 ± 0.48	0.017 *
TMD ^1^	−5.6 ± 3.76	−19.8 ± 2.98	0.004 *

Values are expressed as mean ± SD (*n* = 30). * *p* < 0.05 indicates significant differences using Student’s *t*-test. ^1^ TMD (total mood disturbance) was calculated as (T + D + A + F + C) − V.

**Table 3:** Changes in EEG power spectrum indices in the frontal, parietal, and occipital regions across the animal-assisted activity conditions.

Activity types	**RA ^1^**							
	Fp1 ^2^	Fp2	F3	F4	P3	P4	O1	O2
Video observation	0.16 ± 0.008	0.16 ± 0.007	0.19 ± 0.008	0.18 ± 0.008 ^a^	0.22 ± 0.011 ^a^	0.23 ± 0.011 ^a^	0.21 ± 0.013 ^a^	0.22 ± 0.012 ^a^
Direct observation	0.15 ± 0.008	0.15 ± 0.007	0.18 ± 0.008	0.17 ± 0.008 ^ab^	0.21 ± 0.012 ^ab^	0.21 ± 0.012 ^ab^	0.20 ± 0.012 ^ab^	0.20 ± 0.012 ^ab^
Feeding	0.15 ± 0.005	0.15 ± 0.006	0.16 ± 0.005	0.16 ± 0.005 ^b^	0.18 ± 0.007 ^bc^	0.18 ± 0.006 ^bc^	0.17 ± 0.006 ^bc^	0.17 ± 0.004 ^b^
Grooming	0.15 ± 0.004	0.15 ± 0.005	0.16 ± 0.004	0.16 ± 0.005 ^b^	0.17 ± 0.005 ^c^	0.17 ± 0.005 ^c^	0.16 ± 0.005 ^c^	0.17 ± 0.006 ^b^
*p*-value	0.436	0.319	0.396	0.021	<0.001	<0.001	<0.001	<0.001
Activity types	**RFA**							
	Fp1	Fp2	F3	F4	P3	P4	O1	O2
Video observation	0.05 ± 0.003	0.05 ± 0.003	0.06 ± 0.003	0.06 ± 0.003 ^a^	0.08 ± 0.006 ^a^	0.08 ± 0.006 ^a^	0.08 ± 0.007 ^a^	0.08 ± 0.007 ^a^
Direct observation	0.05 ± 0.002	0.05 ± 0.002	0.06 ± 0.002	0.06 ± 0.002 ^ab^	0.07 ± 0.003 ^ab^	0.07 ± 0.004 ^ab^	0.07 ± 0.003 ^ab^	0.07 ± 0.003 ^b^
Feeding	0.04 ± 0.001	0.05 ± 0.002	0.05 ± 0.001	0.05 ± 0.001 ^b^	0.06 ± 0.002 ^b^	0.07 ± 0.002 ^b^	0.06 ± 0.002 ^b^	0.06 ± 0.001 ^b^
Grooming	0.05 ± 0.001	0.05 ± 0.002	0.05 ± 0.001	0.05 ± 0.001 ^ab^	0.06 ± 0.002 ^b^	0.06 ± 0.002 ^b^	0.06 ± 0.002 ^b^	0.06 ± 0.002 ^b^
*p*-value	0.286	0.207	0.068	0.014	<0.001	<0.001	<0.001	<0.001
Activity types	**RAHB**							
	Fp1	Fp2	F3	F4	P3	P4	O1	O2
Video observation	1.02 ± 0.085	0.98 ± 0.066	1.06 ± 0.059	1.03 ± 0.064	1.30 ± 0.101 ^a^	1.30 ± 0.089 ^a^	1.21 ± 0.111 ^a^	1.24 ± 0.098 ^a^
Direct observation	0.99 ± 0.086	0.93 ± 0.071	1.06 ± 0.082	0.96 ± 0.066	1.20 ± 0.120 ^ab^	1.23 ± 0.103 ^ab^	1.14 ± 0.120 ^ab^	1.11 ± 0.113 ^ab^
Feeding	0.99 ± 0.090	0.97 ± 0.096	0.94 ± 0.052	0.88 ± 0.050	0.95 ± 0.057 ^b^	0.97 ± 0.047 ^bc^	0.92 ± 0.050 ^ab^	0.88 ± 0.039 ^b^
Grooming	0.94 ± 0.065	0.90 ± 0.061	0.94 ± 0.050	0.87 ± 0.050	0.93 ± 0.050 ^b^	0.93 ± 0.035 ^c^	0.86 ± 0.041 ^b^	0.86 ± 0.041 ^b^
*p*-value	0.907	0.848	0.260	0.209	0.005	<0.001	0.013	0.002
Activity types	**RB**							
	Fp1	Fp2	F3	F4	P3	P4	O1	O2
Video observation	0.30 ± 0.011	0.31 ± 0.009	0.33 ± 0.005 ^a^	0.34 ± 0.005	0.34 ± 0.006	0.35 ± 0.005	0.35 ± 0.006	0.35 ± 0.005
Direct observation	0.30 ± 0.010	0.31 ± 0.009	0.33 ± 0.009 ^a^	0.34 ± 0.007	0.35 ± 0.007	0.35 ± 0.007	0.35 ± 0.007	0.35 ± 0.007
Feeding	0.29 ± 0.011	0.30 ± 0.011	0.30 ± 0.006 ^ab^	0.34 ± 0.007	0.35 ± 0.005	0.35 ± 0.004	0.35 ± 0.004	0.36 ± 0.004
Grooming	0.30 ± 0.009	0.31 ± 0.009	0.34 ± 0.006 ^b^	0.34 ± 0.005	0.35 ± 0.005	0.35 ± 0.004	0.36 ± 0.005	0.36 ± 0.005
*p*-value	0.762	0.809	0.044	0.877	0.828	0.736	0.565	0.275
Activity types	**RLB**							
	Fp1	Fp2	F3	F4	P3	P4	O1	O2
Video observation	0.06 ± 0.002	0.06 ± 0.002	0.07 ± 0.002	0.08 ± 0.002	0.09 ± 0.003 ^a^	0.09 ± 0.003 ^a^	0.09 ± 0.004	0.09 ± 0.004 ^a^
Direct observation	0.06 ± 0.002	0.06 ± 0.002	0.07 ± 0.002	0.07 ± 0.002	0.09 ± 0.003 ^ab^	0.09 ± 0.002 ^ab^	0.08 ± 0.003	0.08 ± 0.003 ^ab^
Feeding	0.06 ± 0.002	0.06 ± 0.002	0.07 ± 0.002	0.07 ± 0.002	0.08 ± 0.002 ^ab^	0.08 ± 0.002 ^ab^	0.08 ± 0.002	0.08 ± 0.002 ^ab^
Grooming	0.06 ± 0.002	0.06 ± 0.002	0.07 ± 0.002	0.07 ± 0.002	0.08 ± 0.002 ^b^	0.08 ± 0.001 ^b^	0.08 ± 0.002	0.08 ± 0.002 ^b^
*p*-value	0.368	0.572	0.525	0.188	0.021	0.012	0.085	0.029

Values are expressed as mean ± SD (*n* = 30). ^a,b,c^ Different letters within one column indicate statistical difference (*p* ˂ 0.05). ^1^ RA, relative alpha (8–13 Hz); RFA, relative fast alpha (11–13 Hz); RAHB, ratio of alpha (8–13 Hz) to high beta (20–30 Hz); RB, relative beta (13–30 Hz); RLB, relative low beta (12–15 Hz). ^2^ Fp1, left prefrontal; Fp2, right prefrontal; F3, left frontal; F4, right frontal; P3, left parietal; P4, right parietal; O1, left occipital; O2, right occipital.

## Data Availability

The raw data supporting the conclusions of this article will be made available by the authors upon request.

## Abbreviations

The following abbreviations are used in this manuscript:

POMS	Profile of Mood States
AAA	Animal-assisted activity
AAT	Animal-assisted therapy
EEG	Electroencephalogram
TMD	Total mood disturbance
RA	Relative alpha
RAHB	Ratio of alpha to high beta
RFA	Relative fast alpha
RB	Relative beta
RLB	Relative low beta
